# Overexpression of *ZmSRG7* Improves Drought and Salt Tolerance in Maize (*Zea mays* L.)

**DOI:** 10.3390/ijms232113349

**Published:** 2022-11-01

**Authors:** Xiaotong Wei, Xuhong Fan, Honglin Zhang, Peng Jiao, Zhenzhong Jiang, Xuan Lu, Siyan Liu, Shuyan Guan, Yiyong Ma

**Affiliations:** 1 College of Agronomy, Jilin Agricultural University, Changchun 130118, China; 2 Jilin Academy of Agricultural Sciences, Changchun 130118, China; 3 College of Life Sciences, Jilin Agricultural University, Changchun 130118, China; 4 Joint International Research Laboratory of Modern Agricultural Technology, Ministry of Education, Jilin Agricultural University, Changchun 130118, China

**Keywords:** maize, drought tolerance, salt tolerance, ABA, ROS

## Abstract

Osmotic stress caused by drought and high salinity is the key factor limiting plant growth. However, its underlying molecular regulatory mechanism remains unclear. In this study, we found the stress-related gene Zm00001d019704 (*ZmSRG7*) based on transcriptome sequencing results previously obtained in the laboratory and determined its biological function in maize. We found that *ZmSRG7* was significantly expressed in both roots and leaves under 10% PEG6000 or 150 mM NaCl. Subcellular localization showed that the gene was localized in the nucleus. The germination rate and root length of the *ZmSRG7* overexpressing lines were significantly increased under drought or salt stress compared with the control. However, after drought stress, the survival rate and relative water content of maize were increased, while the water loss rate was slowed down. Under salt stress, the Na^+^ concentration and Na^+^: K^+^ ratio of maize was increased. In addition, the contents of antioxidant enzymes and proline in maize under drought or salt stress were higher than those in the control, while the contents of MDA, H_2_O_2_ and O_2_^−^ were lower than those in the control. The results showed that the *ZmSRG7* gene played its biological function by regulating the ROS signaling pathway. An interaction between ZmSRG7 and the Zmdhn1 protein was found using a yeast two-hybrid experiment. These results suggest that the *ZmSRG7* gene can improve maize tolerance to drought or salt by regulating hydrogen peroxide homeostasis.

## 1. Introduction

Maize (*Zea mays* L.) is widely cultivated around the world as a multiple-use crop [1]. Plants are exposed to various complex and variable environmental factors from the moment their seeds are planted. Environmental conditions that are not conducive to plant growth and development are collectively referred to as stress [2]. Stress includes biotic stress and abiotic stress. Abiotic stresses such as high salt and drought affect 10% of the world’s arable land, resulting in yield loss of important crops such as maize, rice and wheat by more than 50% [3]. A previous study showed that under long-term water deficit and high salt osmotic stress, the growth, development, yield and quality of maize were affected to different degrees, leading to reduction in yield and quality. Therefore, it is essential to resist abiotic stress and increase maize yield. Plants have evolved a variety of defense mechanisms to adapt to adversity: different signaling pathways in the plant body regulate gene expression series in energy metabolism, ion and water transport, protein degradation, and active oxygen removal of changes in molecular, cellular, physiological, and biochemical levels to improve survival under adversity [4,5]. In recent years, more and more drought resistance genes have been identified. Guo et al. [6] in a genome-wide association analysis (GWAS) of 507 rice samples, identified 470 associated loci, of which 437 were co-localized with previously reported QTLs for drought resistance traits. *OsPPI5* was found to be closely related to one of the core traits, and its important drought-resistance function was demonstrated. Overexpression of *ZmWRKY65*, for example, can improve stress tolerance in transgenic *Arabidopsis* [7]. *ZmbZIP4* helps maize survive stress by regulating Abscisic acid (ABA) production and root growth [8]. Zhou et al. [9] reported that rice *OsSTRK1* significantly increased CatC activity by phosphorylating Tyr210 tyrosine residue of catalase CatC, with which it interacts, thereby regulating ROS (reactive oxygen species) homeostasis and improving salt tolerance and rice yield.

ROS are a necessary chemical component of aerobic life [10]. Plants have evolved an antioxidant defense system to mop up chemicals such as ROS in order to survive under stress [11]. These ROS are activated by stress, resulting in the production of peroxidase (POD), superoxide dismutase (SOD), glutathione peroxidase (GPx), and catalase (CAT) [12,13]. The proline content of the osmotic protective material increases with stress time, which benefits the integrity of the biofilm and cell turgor [14]. Malondialdehyde (MDA) buildup damages the plant’s cellular structure, causing cell rupture [15]. Numerous studies found that, compared to drought-sensitive maize inbred lines, drought-resistant maize inbred lines had higher relative water content, antioxidant enzyme activity, and proline content, while the latter had lower levels of H_2_O_2_, MDA, relative electrical conductivity, and degrees of cell damage [16,17,18,19]. Qiu et al. [20] demonstrated that overexpression of *TaASR1-D* in transgenic wheat improves its resistance to oxidative stress. Under drought stress, the activities of the transgenic lines’ SOD, CAT, and GPx activities were frequently higher than those of wild-type (WT) lines.

The most widely distributed soluble cation in saline soils is sodium ion (Na^+^), which harms plants primarily by creating prolonged osmotic stress and ionic toxicity [21]. Sodium ions enter plant roots in saline circumstances and are transferred to aboveground tissues by transpiration flow. Excessive Na^+^ movement from root to stem and photosynthesis-induced Na^+^ accumulation, on the other hand, are detrimental to crops, resulting in lower photosynthetic carbon absorption and even yield reduction [22]. Plants have evolved a variety of ways to avoid the harmful effects of sodium in high Na^+^ environments [21]. For example, Na^+^ is barred from transpiration streams, and these mechanisms are mostly mediated by ion transporters, particularly those that prefer sodium ions [23]. Previous studies have shown that the *NHX* and *HKT1* families of genes, which encode selective Na^+^ transporters, are essential for maintaining Na^+^ homeostasis and salt tolerance [24]. ABA is one of the most important hormones involved in stress signal transduction. Salt and drought stress can promote the accumulation of ABA in plants. Ye et al. [25] found that salt stress and ABA treatment induced the expression of the *MpSnRK2.10* gene, and overexpression of this gene alleviated salt stress as the limitation of apple growth. *ZmFLZ25* is thought to be involved in ABA signal transduction in plants because the ectopic overexpression of *ZmFLZ25* in *Arabidopsis* results in hypersensitivity to exogenous ABA and increases the expression of ABA-induced genes. This is supported by the interaction of *ZmFLZ25* and the ABA receptor [2]. Both the ABA-dependent and ABA-independent signaling pathways see an uptick in gene expression in times of stress [26]. Important signaling proteins in plants, such as transcription factors, phosphatases, and protein kinases, transmit signals that upregulate the expression of stress-resistance genes. Zong et al. [27] demonstrated that the sensitivity of transgenic cotton seed germination, the seedling development stage, and stomatal movement to a certain concentration of ABA was greater than that of WT cotton, implying that *ABP9* may act as a response signal of the ABA signaling pathway in early plant growth. Wang et al. [28] discovered that under salt and drought stress, *ZmHsf08* adversely regulates many ABA-sensitive genes.

Maize is one of the most important global crops. To ensure food security, it is of great importance to cultivate new maize varieties with strong abiotic stress resistance. In this study, a *ZmSRG7* overexpression vector was constructed and transformed into maize. The tolerance of transgenic maize plants to oxidative stress, osmotic stress, drought stress and salt stress was enhanced. In addition, we found that *ZmSRG7* played a role in this by enhancing the antioxidant system and ABA-mediated ROS signal transduction. Although a number of genes related to abiotic stress have been discovered and isolated, their function in maize has not been well investigated. Given that abiotic stress is currently threatening maize output, it is both theoretically and practically vital to investigate and identify the relevant genes involved in maize stress resistance using appropriate molecular biology methodologies.

## 2. Results

### 2.1. Induced Expression of ZmSRG7 under Stress Conditions

**Figure 1:**
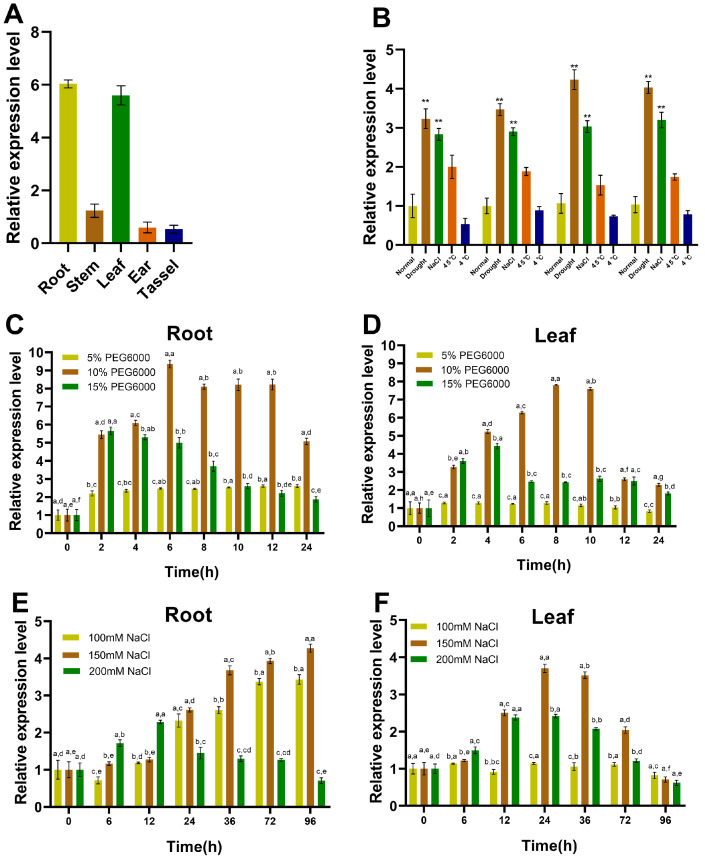
Expression analysis of *ZmSRG7*. (**A**) tissue site expression analysis of *ZmSRG7*. (**B**) response of *ZmSRG7* to abiotic stress. (**C**–**F**) expression of *ZmSRG7* in roots and leaves. Values are mean ± SD of three biological replicates. Bars with different letters are significantly different at *p* < 0.05 according to Duncan’s multiple range tests. *p* < 0.05 (**).

The expression of the *ZmSRG7* gene, isolated from maize inbred line B73, in roots, stems, leaves, ears, and tassels was detected using qRT-PCR. According to the data, *ZmSRG7* expression was found to be higher in roots and leaves but lower in tassels (Figure 1A). The complete seedlings were sampled 0, 2, 4, and 12 h after hydroponic treatment under 10% PEG6000 solution, 150 mM NaCl solution, 45 °C, and 4 °C, respectively, to determine the response of the *ZmSRG7* gene to abiotic stress. The results revealed that *ZmSRG7* was highly activated by drought and salt stress (Figure 1B). Next, we evaluated the expression of *ZmSRG7* in roots and leaves following 5%, 10%, and 15% PEG6000 treatments at 0, 2, 4, 6, 8, 10, 12, and 24 h to characterize its response to drought stress (Figure 1C,D). Even after 2 h of treatment with 10% PEG6000, *ZmSRG7* expression remained highly elevated in the roots (Figure 1C). *ZmSRG7* expression was high in leaves for 10 h following a 2 h treatment with 10% PEG6000 (Figure 1D). Next, to investigate the response of *ZmSRG7* to salt stress, the expression levels of *ZmSRG7* in roots and leaves were measured following treatments with 100 mM, 150 mM, and 200 mM NaCl for 0, 6, 12, 24, 36, 72, and 96 h (Figure 1E,F). The 100 mM NaCl treatment for 24 h stimulated *ZmSRG7* expression in roots, which was then augmented by the 150 mM NaCl treatment for 36, 72, and 96 h (Figure 1E). Leaf expression was induced by 150 mM NaCl for 24 and 36 h (Figure 1F).

In conjunction with these findings, we determined that the expression level of *ZmSRG7* in roots was greatest under stress, and that gene expression was greatest under 10% PEG6000 and 150 mM NaCl stress. Consequently, this condition served as the stress condition in the subsequent tests. The results of these tests suggested that abiotic stress up-regulated the *ZmSRG7* gene.

### 2.2. Subcellular Localization of ZmSRG7

**Figure 2:**
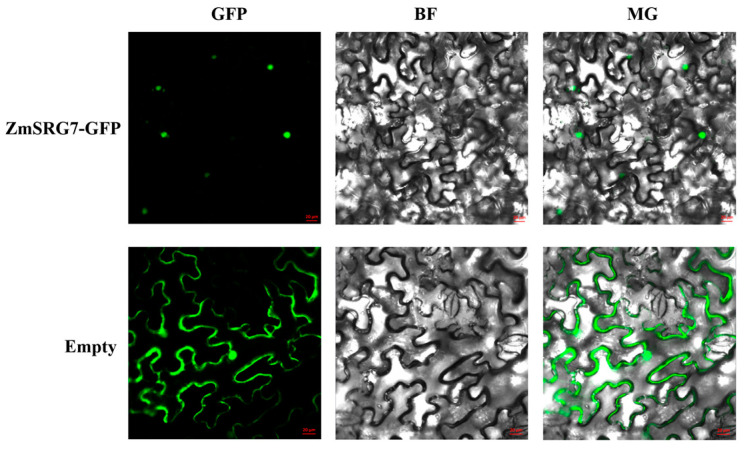
Analysis of *ZmSRG7* protein subcellular localization in tobacco cells. The scale bar represents 20 μm.

Transient expression of ZmSRG7-GFP was performed in tobacco leaves with the purpose of observing the subcellular localization of *ZmSRG7*. The green fluorescence signal produced by the control vector GFP was visible everywhere. On the other hand, the ZmSRG7-GFP signal was only seen on the nucleus of the cell (Figure 2). This finding was in line with what was anticipated.

### 2.3. Overexpression of ZmSRG7 in Transgenic Maize Can Improve Maize Osmotic and Drought Stress Tolerance

**Figure 3:**
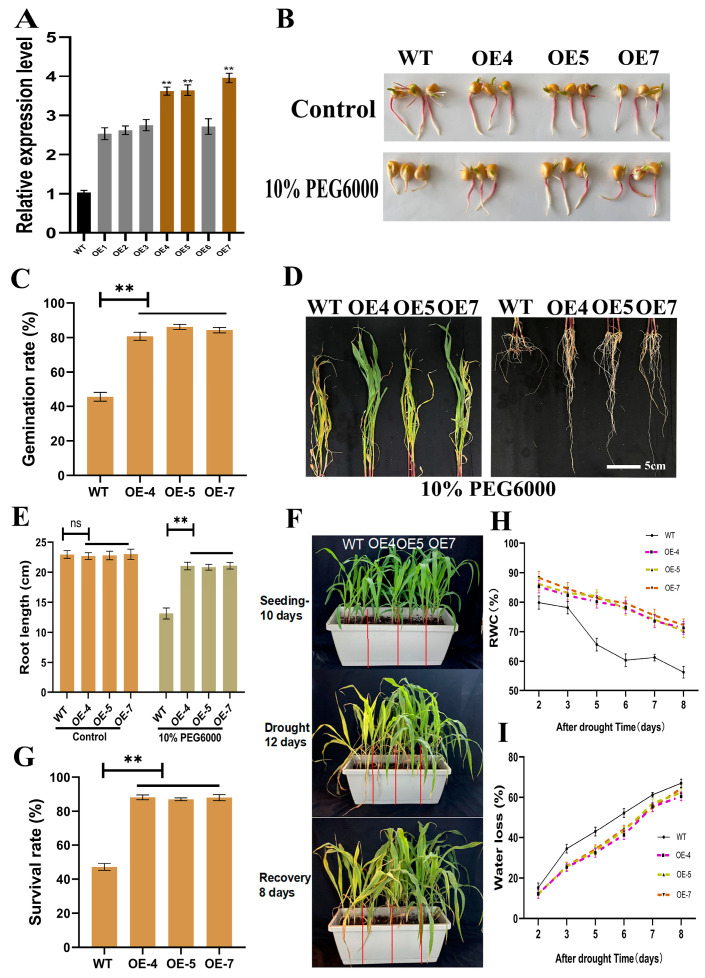
Overexpression of *ZmSRG7* endows plants with drought resistance. (**A**) expression level of transgenic lines (OE1-7). (**B**,**C**) germination of transgenic lines. (**D**–**F**) phenotype and root length statistics of 10% PEG6000 under osmotic stress. (**G**) natural drought phenotype. (**H**,**I**) survival rate, RWC and water loss rate under drought stress. Values are mean ± SD of three biological replicates. Bars with different letters are significantly different at *p* < 0.05 according to Duncan’s multiple range tests. Non-significance (ns), *p* < 0.05 (**).

To verify gene function, we created a *ZmSRG7* overexpression vector and used an agrobacterium-mediated method to transform *ZmSRG7* into the maize inbred line H8204, yielding seven transgenic lines (OE1-7). Three transgenic lines with high expression levels (OE-4, OE-5, OE-7) were tested in the T_3_ generation (Figure 3A). In order to verify the function of *ZmSRG7* under drought stress, plants were treated in a solution containing 10% PEG6000. The results showed that OE and WT seeds were able to germinate, and the germination rate of OE increased by 84.12% (Figure 3B,C). Further, 3-day-old OE and WT seedlings were hydroponically grown for 7 d in a solution containing 10% PEG6000 before the lengths of their roots were measured. The transgenic root lengths increased by 59.44% compared to the WT (Figure 3D,E). The longer relative root lengths in transgenic maize seedlings suggest that *ZmSRG7* overexpression enhanced transgenic maize seedling growth under osmotic stress. All the plants displayed damaged phenotypes under osmotic stress after 7 d of treatment with 10% PEG6000, whereas the WT lines displayed more severe wilting and yellowing (Figure 3D). 

To determine the OE lines’ tolerance to water scarcity, WT and OE seedlings were planted in the same container and allowed to grow normally for 10 d. When water was cut off for 5 d, the leaves of WT appeared to roll and then began to wither, whereas the leaves of OE lines appeared to roll but remained green (Figure 3F). OE lines recovered their leaf shape faster than WT lines during the rehydration process. The survival rate, relative water content, and rate of water loss were all measured 8 d after rehydration. OE lines had an 86% higher survival rate than WT lines (Figure 3G). The RWC (relative water content) for WT lines was obviously lower than for overexpressed lines, but the opposite was found for the rate of water loss (Figure 3H,I). As a result, maize seedlings with increased *ZmSRG7* expression were found to have better water retention properties when dehydrated. These findings suggested that maize *ZmSRG7* overexpression improved osmotic stress and drought stress tolerance.

### 2.4. Overexpression of ZmSRG7 in Transgenic Maize Can Improve the Salt Tolerance of Maize

**Figure 4:**
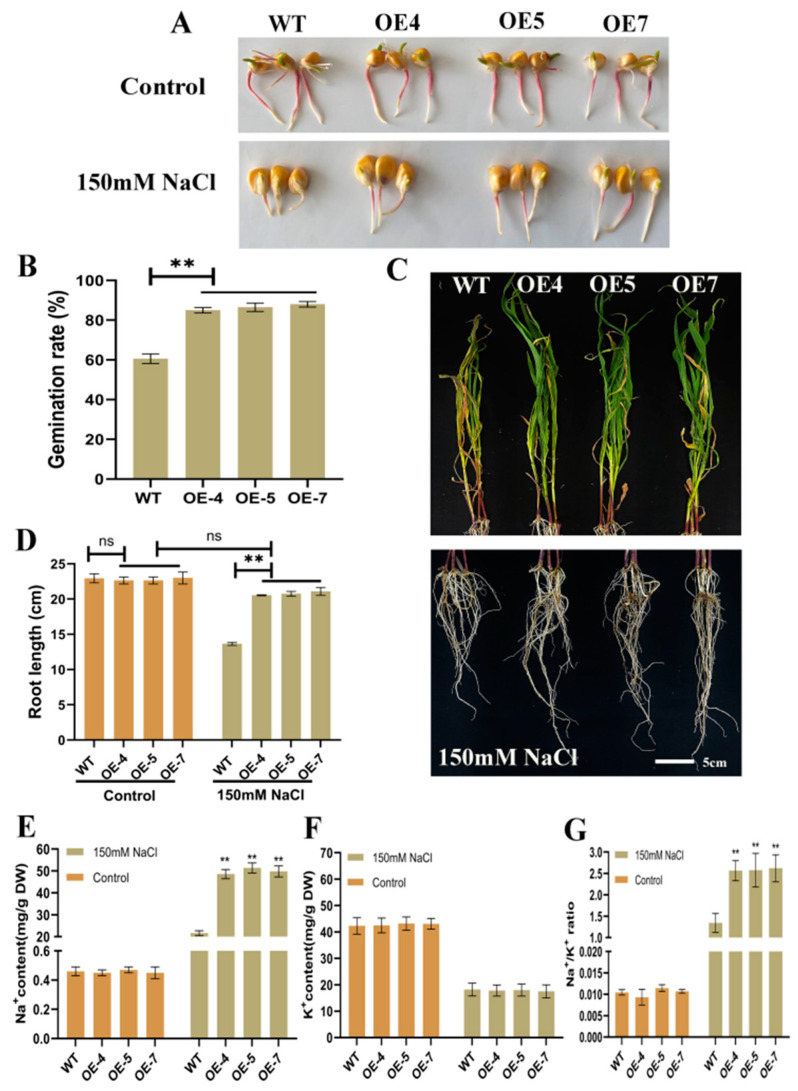
Salt tolerance of *ZmSRG7* gene. (**A**,**B**) germination of WT and OE under salt stress. (**C**,**D**) root length and leaf phenotype under salt stress. (**E**–**G**) Na^+^, K^+^ content and Na^+^: K^+^ ratio under salt stress. Values are mean ± SD of three biological replicates. Bars with different letters are significantly different at *p* < 0.05 according to Duncan’s multiple range tests. Non-significance (ns), *p* < 0.05 (**).

The germination rates of OE and WT seeds in 150 mM NaCl were compared to characterize the salt tolerance of overexpressed *ZmSRG7*, and the transgenic seeds showed a 42.74% increase (Figure 4A,B) (to enable comparison, a single set of untreated germination maps were shared by salt stress and drought stress). There were apparent supporting roots under the stem, showing that high salinity reduced the root lengths of WT lines, which grew by 52.38% compared to WT lines, but there was no significant change in leaf growth (Figure 4C,D). To investigate how *ZmSRG7* improves salt tolerance in transgenic maize seedlings, we compared the Na^+^ and K^+^ concentrations in the roots of WT and OE lines. After being subjected to salt, both WT and OE lines showed an increase in Na^+^ content and a decrease in K^+^ concentration (Figure 4E,F). Under both the control and salt stress conditions, there was no discernible difference in K^+^ content between WT and OE lines (Figure 4F). However, OE lines collected more Na^+^ and had a higher Na^+^: K^+^ ratio than WT lines, which were treated with NaCl (Figure 4E,G). These findings therefore suggest that overexpression of the *ZmSRG7* gene can resist salt stress.

### 2.5. Overexpression of ZmSRG7 in Transgenic Maize Can Improve the Antioxidant Capacity of Maize

**Figure 5:**
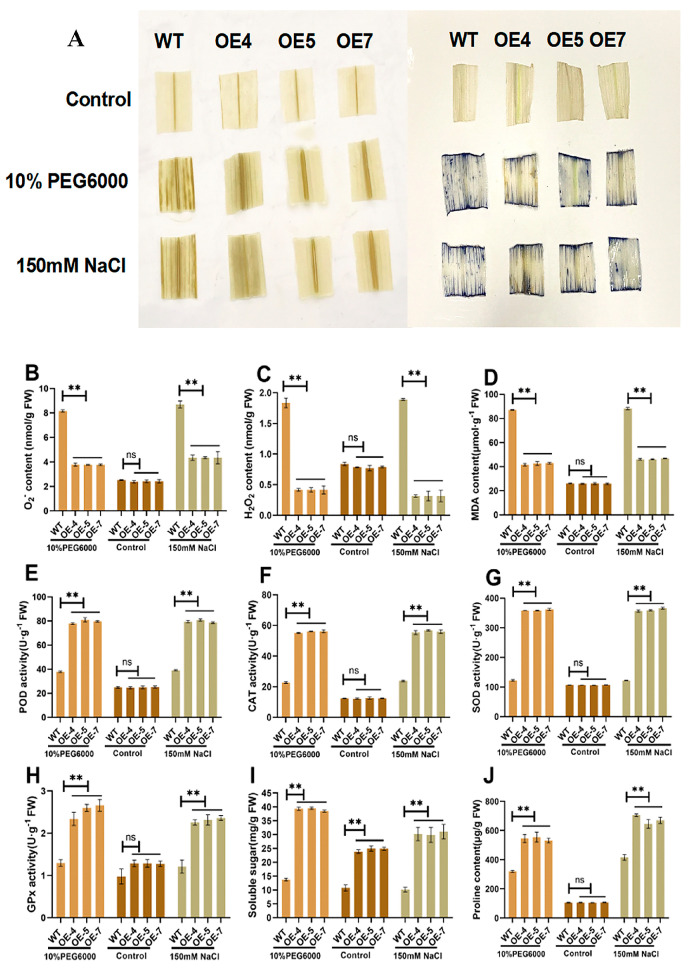
Oxidative stress of *ZmSRG7*. (**A**) DAB and NBT staining. (**B**,**C**) O_2_^−^ and H_2_O_2_ content analysis. (**D**) MDA content. (**E**–**H**) analysis of antioxidant enzyme activity (POD, CAT, SOD, GPx). (**I**) soluble sugar content. (**J**) proline content. Values are mean ± SD of three biological replicates. Bars with different letters are significantly different at *p* < 0.05 according to Duncan’s multiple range tests. Non-significance (ns), *p* < 0.05 (**).

Next, 3,3′-diaminobenzidine (DAB) and Nitroblue tetrazolium (NBT) staining methods were used to determine the antioxidant capacity of the overexpressed *ZmSRG7* gene. The results showed that, under salt or drought conditions, the leaves of WT maize were stained with DAB and NBT, and the degree of staining was deep. Maize overexpressing *ZmSRG7* was lighter in color than the WT (Figure 5A). This may be because the overexpression of the *ZmSRG7* gene reduces the generation of H_2_O_2_ and thus reduces the accumulation of ROS. It was preliminarily concluded that the *ZmSRG7* gene has a certain antioxidant ability, and that it reduces oxidative stress. In order to further clarify the causes of decreased ROS accumulation in *ZmSRG7* maize overexpression lines, the expression of oxidative factors and antioxidant factors in maize leaves was detected after stress treatment. When treated with NaCl or PEG, the expression of H_2_O_2_ and O_2_^−^ decreased in the *ZmSRG7*-overexpressing lines compared with the control (Figure 5B,C). The activity of ROS-scavenging-related enzymes was measured. As shown in Figure 5E–H, after stress, the enzyme activities of POD, CAT, SOD and GPx in maize overexpression lines were significantly higher than those in the WT. However, there was no significant difference between the overexpressed *ZmSRG7* gene without stress treatment and the control, which was consistent with the staining results. The results showed that oxidative stress damage was induced in maize after stress, and overexpression of the *ZmSRG7* gene was able to improve the activity of ROS-scavenging-related enzymes and promote the ROS scavenging ability of cells under stress treatment conditions. It is well known that ROS can cause damage to a variety of biological macromolecules in cells, such as lipids. Polyunsaturated fatty acids of membrane lipids are susceptible to ROS-induced peroxidation, and produce various aldehydes, enals and hydroxyl alkenes, including the cytotoxic compound MDA [29]. To further determine the degree of oxidative damage in each line, we measured the content of MDA in each line. As shown in Figure 5D, MDA content in maize overexpression lines was significantly lower than that in the WT after both the untreated and stress treatments. These results indicated that *ZmSRG7* may affect the intracellular REDOX balance and reduce oxidative stress damage.

Soluble sugars can effectively reduce cellular water potential, and plants can respond to stress by reducing intracellular water potential. Secondly, free proline in plants also has a protective effect on cells under stress [30]. Therefore, the soluble sugar and proline contents of the overexpressing lines were examined (Figure 5I,J). The results showed that the soluble sugar and proline contents of the *ZmSRG7* overexpressing lines were significantly higher than those of the WT. These results indicated that the *ZmSRG7* gene may resist stress by regulating ROS and osmoregulatory substances, thus promoting the growth of maize.

### 2.6. Related Gene Expression Analyses of Transgenic Maize

**Figure 6:**
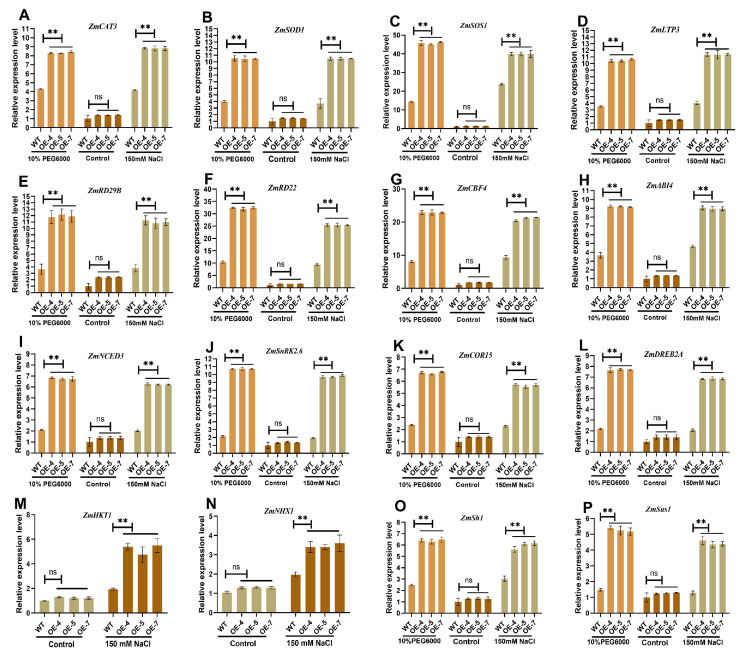
Related gene expression situation. (**A**–**H**) expression analysis of ROS pathway related genes. (**I**–**L**) expression of genes related to ABA pathway. (**M**,**N**) expression analysis of sodium ion transporter gene. (**O**,**P**) expression analysis of sugar synthase related genes. Values are mean ± SD of three biological replicates. Bars with different letters are significantly different at *p* < 0.05 according to Duncan’s multiple range tests. Non-significance (ns), *p* < 0.05 (**).

We analyzed the expression patterns of marker genes involved in ROS to better characterize the functional mechanism of *ZmSRG7*. qRT-PCR was used to investigate the expression of ROS-scavenging and antioxidant genes in *ZmCAT3*, *ZmSOS1*, *ZmSOD1*, *ZmLTP3*, *ZmRD29B*, *ZmRD22*, *ZmCBF4*, and *ZmABI4* [31]. When WT and OE lines were treated with 10% PEG6000 and 150 mM NaCl, eight marker genes were activated, and the expression levels of these genes in OE lines were noticeably higher than those in WT lines (Figure 6A–H). The detection of these indicators fully proved that the *ZmSRG7* gene can resist stress through regulating the ROS signaling pathway.

Next, we analyzed the gene expression of *COR15* and *DREB2A*, which are involved in the ABA-independent pathway, and *NCED3*, a well-known marker of the ABA-dependent pathway. Transgenic materials treated with 10% PEG6000 and 150 mM NaCl showed significant changes in the expression of *NCED3* and *SnRK2.6* in the ABA-dependent pathway compared to controls (Figure 6I,J). There was also a notable shift in the expression of *COR15* and *DREB2A* (Figure 6K,L). These results indicated that overexpression of the *ZmSRG7* gene may also participate in the regulation of key genes involved in the ABA pathway to resist stress.

Furthermore, two genes involved in transporting sodium ions, *ZmHKT1* and *ZmNHX1*, were found to be highly expressed. After being exposed to salt, *ZmHKT1* and *ZmNHX1* expression levels increased, and OE lines had higher levels of these genes than WT lines did (Figure 6M,N). We found that the leaves of the OE lines expressed the glycosynthase-related genes *ZmSh1* and *ZmSus1*, which is significant because sugar tolerance is essential for plant abiotic stress, and soluble sugar content was found to have increased. The results indicated that *ZmSh1* and *ZmSus1* expression were elevated in response to stress (Figure 6O,P).

### 2.7. Comparison of Yeast Growth under Drought and Salt Stress

**Figure 7:**
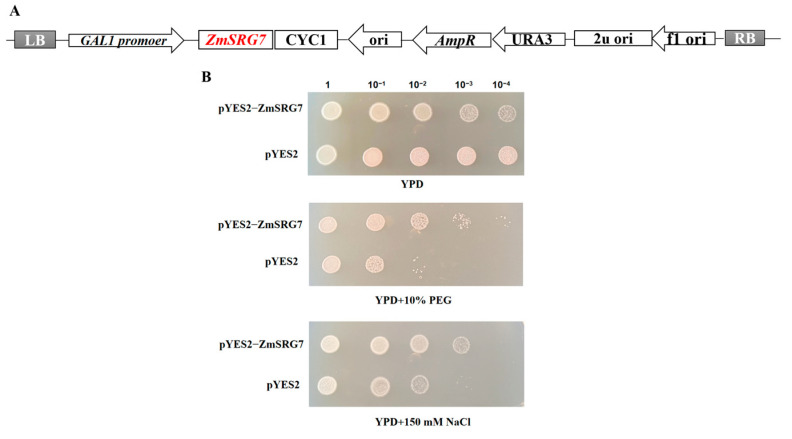
Growth of *ZmSRG7* in yeast. (**A**) construction of yeast overexpression vector of pYES2:ZmSRG7. (**B**) growth of yeast under 10% PEG6000, 150 mM NaCl stress.

Next, a pYES2-ZmSRG7 yeast overexpression vector was created (Figure 7A). Under drought and salt stress circumstances, there was no significant difference in the growth of INVSC1 (pYES2-ZmSRG7) and INVSC1 (pYES2) (Figure 7B). Furthermore, the expression of the *ZmSRG7* gene in yeast had no effect on normal yeast growth. Under drought stress, INVSC1 (pYES2-ZmSRG7) and INVSC1 (pYES2) were inoculated at the same density on SC-URA solid medium containing 2% galactose at the original concentration and 10 dilutions. After they were diluted 100 times, the number of yeast colonies of INVSC1 (pYES2-ZmSRG7) was found to be greater than that of INVSC1 (pYES2). After they were diluted 1000 and 10,000 times, the differences between INVSC1 (pYES2) and INVSC1 (pYES2-ZmSRG7) became more apparent. INVSC1 (pYES2-ZmSRG7) had essentially little growth, whereas INVSC1 (pYES2-ZmSRG7) still had a substantial amount of growth. The results demonstrated that expressing the exogenous *ZmSRG7* gene increased transgenic yeast’s drought tolerance considerably. In both yeast species, NaCl stress was equivalent to drought stress. However, INVSC1 (pYES2-ZmSRG7) was more prominent in point culture, and INVSC1 (pYES2) was much lower than INVSC1 (pYES2-ZmSRG7) after being diluted 100, 1000, and 10,000 times (Figure 7B). These results demonstrated that transgenic yeast was more resistant to salt stress than non-transgenic yeast.

### 2.8. One-to-One Validation of ZmSRG7 Interacting Proteins

To avoid reporter gene expression caused by the inserted target fragment, which would have interfered with the screening of interacting proteins, it was important to determine whether the pGBKT7-ZmSRG7 recombinant vector possessed autoactivation capability. pGBKT7-ZmSRG7 + pGADT7-dhn1 (experimental group), pGBKT7-53 + pGADT7-T (positive control), and pGBKT7-Lam + pGADT7-T (negative control) plasmids were transfected into yeast competent (Y2H Gold) cells. By treating the two nutrient-deficient media, the autoactivation was confirmed. The results demonstrated that the experimental group ZmSRG7-BD + Zmdhn1-AD, the negative control pGBKT7-Lam + pGADT7-T, and the positive control pGBKT7-53 + pGADT7-T were all able to grow normally on ditrophic media (-Leu/-Trp). In the four-deficient medium containing X-α-Gal chromogen (-Ade/-Leu/-Trp/-His), only the experimental group ZmSRG7-BD + Zmdhn1-AD and the positive control pGBKT7-53 + pGADT7-T were able to grow normally and become blue. Finally, the yeast proteins ZmSRG7 and Zmdhn1 were found to interact.

## 3. Discussion

Abiotic stress, such as drought, high salt and low temperature, seriously affected the growth of maize, and is the main factor limiting the yield of maize [32]. Therefore, it is a priority of scientific research to explore the functional genes of maize related to stress adversity. Based on maize stress transcriptome sequencing data completed in the laboratory (NCBI: PRJNA793522), the *ZmSRG7* gene with significantly up-regulated expression was selected (Figure S1). Studies have shown that this gene is highly expressed in roots and leaves in response to drought and salt stress (Figure 1), and is a dual resistance gene, so we named it *ZmSRG7* (stress-related gene, mapping chromosome 7, SRG7). Muthusamy et al. [33] found that *BrEXLB1* (Brassica rapa Expansin-Like B1) is involved in root development, the drought stress response, and seed germination. Therefore, the seed germination rate under stress is very important for plant growth and development. In this study, the overexpression of *ZmSRG7* was found to significantly enhance the drought resistance and salt tolerance of plants, and the germination rate of transgenic seeds was found to increase by 84.12% and 42.74% under drought and salt stress, respectively (Figure 3B,C and Figure 4A,B). The root system is an important organ for crops to absorb nutrients and water, and the cultivation of a developed and robust root system is an important means for most crops to realize their yield potential in high-yield cultivation. Gautam et al. [34] found that the *LBL1* mutant *LBL-rgd1* played a role in maize root development, and compared the root phenotype with the WT at 7 d after germination. Furthermore, the taproot of *LBL1-rgd1* was found to be about 72.61% longer than that of the WT. In this study, the root lengths of transgenic plants under drought and salt stress increased by 59.44% and 52.38% compared with the WT, respectively (Figure 3D and Figure 4C). Under natural drought conditions, WT leaves showed withered and yellowing phenotypes, and hardly changed after rehydration, while the transgenic plants were green during this period, and were able to grow normally after rehydration. Furthermore, the RWC of the transgenic plants was higher than that of the WT, while the opposite was true for the water loss rate (Figure 3F,I).

It is often observed that there is no strong correlation between sodium content and salt tolerance [35]. Under high salinity, plants can isolate Na^+^ into vacuoles against concentration gradients by Na^+^/H^+^ antiporter located in their vacuolar membranes and plasma membranes, or reverse transport Na^+^ out of cells to maintain intracellular ion balance [36]. In addition, the SOS signaling system also plays a very important role in regulating ion homeostasis and improving plant salt tolerance. This signaling pathway is closely related to the salt stress response, and includes three major proteins, SOS1, SOS2 and SOS3. The *SOS1* gene encodes a Na^+^/H^+^ antiporter at the plasma membrane [37]. Roots play an important role in controlling sodium absorption and transport over long distances, and *ZmSRG7* is highly expressed in roots. In this study, the amounts of Na^+^ and K^+^ in roots after salt treatment were examined, and the buildup of Na^+^ in OE lines was found to be larger than in WT (Figure 4E–G), possibly due to lower expression of the sodium repelling gene (Figure 6N). We detected significant expression of the *SOS1* gene in the overexpressed lines (Figure 6C), so we hypothesized that the mechanism of salt tolerance involves transporting excessive Na^+^ out of the cell by the Na^+^/H^+^ antiporter to maintain normal homeostasis. As a result of the enhanced expression of *HTK1* (Figure 6M), transgenic lines’ salt tolerance may have been improved. This is consistent with the research results of Zhang et al. [23,24]. It is well known that high salt and drought can cause osmotic stress. In an osmotic stress environment, soluble sugar can effectively vitrify the liquid around chloroplasts to reduce the water potential of cells, thus playing a protective role in plants. The soluble sugar content of the *ZmSRG7* overexpression lines under salt and drought stress was significantly higher than that of the WT and the high expression level of sugar-synthetase-related genes, indicating the enhanced tolerance of the transgenic lines to osmotic stress (Figure 5I and Figure 6O,P). 

In order to further determine the function of the *ZmSRG7* gene, we tested its physiological and biochemical indexes. ROS are the product of the incomplete reduction of oxygen molecules, and are highly toxic [10]. Under abiotic stress, ROS can not only destroy the structure and function of cells, but also be an important regulator of signal transduction [16]. Stress causes osmotic stress, oxidative stress, and hazardous chemical buildup [1]. Jiao et al. [31] showed that overexpression of *ATHB-6* improved the drought tolerance of maize and mediated the ROS signaling pathway and ABA-dependent pathway. As a result, in this study, we took measurements of the transgenic plants’ physiological and biochemical properties. Transgenic plants were found to have lower ROS accumulation and MDA content than WT lines under normal and stressful circumstances (Figure 5A,D). Further, we found that transgenic plants expressed more ROS-related genes than WT plants, implying that the *ZmSRG7* gene is engaged in the ROS signaling pathway. To avoid injury, plants boost the activity of antioxidant enzymes (POD, SOD, CAT, GPx) in their bodies when they are stressed (Figure 5E–H). In this study, the antioxidant enzyme activity of OE lines was always higher than that of the WT, while the proline concentration was also always higher than that of the WT (Figure 5J). This is consistent with the research results of Qiu et al. [20]. Under drought and salt treatments, greater sugar synthase gene expression and soluble sugar concentration boosted OE lines’ osmotic stress tolerance (Figure 5I, Figure 6O,P). To investigate if the ABA signaling system is involved in plant adaptation to stress, we evaluated the expression levels of ABA-related genes. Under normal and treated circumstances, the transcription levels of ABA-up-regulated genes in OE lines were always higher (Figure 6I–L). These findings show that the overexpression of *ZmSRG7* improves ABA signal transduction in maize, and that *ZmSRG7* may play a role in ABA production and signaling.

**Figure 8:**
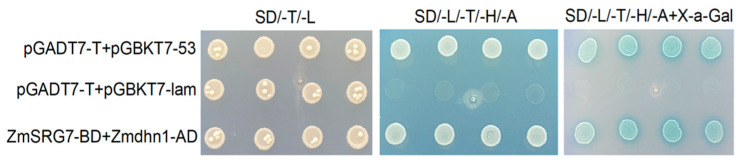
Validation of interaction between *ZmSRG7* and *Zmdhn1* in yeast. pGBKT7-53 + pGADT7-T: positive control; pGBKT7-Lam + pGADT7-T: negative control; pGBKT7-ZmSRG7 + pGADT7-dhn1: experimental group.

Abiotic stress is harmful to plants in many ways, from impacting plant growth to affecting the internal environment of various plant cells. Long-term selective evolution requires that the genes generated in plants in response to stress be related to one another in order to coordinate the regulation, resistance, and repair of stress damage. Interaction gene screening is a method for investigating the internal gene network of the plant complex stress response. Through this method, it has been found that the uptake and transport of aluminum in *Arabidopsis*, as a plasma membrane transporter, requires the cooperation of the malate transporter ALMT1, due to NIP1, a member of the aquaporin (AQP) family [38]. The *ZmSRG7* protein is subcellularly localized in the nucleus and has the ability to directly regulate maize water balance under stress conditions via its expression level (Figure 2). When plants are subjected to abiotic stress, their adaptation mechanism is governed by multiple complex regulatory networks. The yeast double hybrid experiment technology was used in this study to verify the ZmSRG7 protein and Zmdhn1 protein one-on-one, and the results showed that these proteins interacted in yeast (Figure 8). Zmdhn1 is a member of the DHN dehydration protein family, and the protein encoded by Zmdhn1 has functions such as oxidative stress tolerance, low temperature tolerance, and an internal signal transduction mechanism that is related to plant tolerance.

In general, this study successfully excavated the stress-related *ZmSRG7* gene through completed abiotic stress transcriptome sequencing data of maize, and studied the function and mechanism of this gene by overexpressing it. The results showed that the expression of the *ZmSRG7* gene was not tissue-specific, but it was highly expressed in roots and leaves, and was able to be induced by salt and drought stress. The ZmSRG7 protein is mainly localized in the nucleus. Our results suggest that the overexpression of *ZmSRG7* enhances the stress tolerance of transgenic maize plants through improving the antioxidant system and ABA-mediated ROS signaling pathway, which jointly play a response function in salt and drought stress. In addition, a yeast double hybrid experiment was used to verify the interaction relationship between ZmSRG7 and Zmdhn1, which provides directions for future research. In summary, our study shows that *ZmSRG7* is a dual resistance gene and that its overexpression improves drought and salt tolerance in maize, which is a major advance in crop gene breeding research.

## 4. Materials and Methods

### 4.1. Plant Materials and Growth Conditions

Maize inbred line H8204 was used as experimental material. The maize was cultured in a room with long periods of sunshine (16 h of light/8 h of darkness) at 25 °C. Surface sterilized seeds were germinated on 1/2 MS liquid medium with or without 10% PEG6000 and 150 mM NaCl. In order to evaluate the tolerance of transgenic plants to osmotic stress, the root length was determined at 14 d of growth in hydroponics with or without 10% PEG6000 and 150 mM NaCl. To evaluate drought tolerance, three OE lines and WT lines were grown in the same pot, soil and vermiculite were added (3:1), and natural drought lasted for 20 d after 10 d of growth. Then, all the plants were irrigated for 8 d, and their survival rate, relative water content and water loss rate were calculated. To analyze the expression patterns of related genes, 3-week-old seedlings were transferred to 150 mM NaCl and 10% PEG6000 for 12 h.

### 4.2. Construction of Plasmids and Genetic Transformation

The encoding sequence for *ZmSRG7* (Zm00001d019704) was introduced into the pCAMBIA3301 plasmid, which was driven by the maize 35S promoter. Genetic transformation of maize was performed as described [31]. Experiments were carried out using seeds of transgenic maize from homozygous T_3_ generation. 

### 4.3. Tobacco Transient Transformation and Subcellular Localization Vector Construction

The plasmid from the recombinant vector pCAMBIA1302-Ubi-ZmSRG7-GFP was successfully introduced into Agrobacterium EHA105 [31]. A 2.5 mL syringe was used to inject bacterial solution into the back of 6-week-old Nicotiana benzoi young leaves. The green fluorescence of the leaves transformed with recombinant plasmid was observed under a laser confocal microscope after incubation at 22 °C and 16 h light/8 h dark for 24–48 h to determine the position of ZmSRG7 protein in the cells.

### 4.4. Physiological Indices Measurements

The contents of hydrogen peroxide, MDA, soluble sugar and proline, as well as the activities of SOD, CAT, GPx and POD, were detected using the detection kit [18,39]. The content of superoxide anion was determined with a detection kit [40]. The RWC values and water loss rate were determined based on the above method [41].

### 4.5. Histochemical Staining

In order to detect the endogenous hydrogen peroxide levels under normal and stress conditions, corn seedlings at the age of three weeks were added with 10% PEG6000 and 150 mM NaCl in 1/2 MS medium, followed by DAB and NBT staining [41].

### 4.6. Na^+^ and K^+^ Concentrations Are Determined

For analysis of Na^+^ and K^+^ contents in roots under normal and high salinity conditions, the 3-week-old seedlings were treated with or without 150 mM NaCl for 7 d. The contents of Na^+^ and K^+^ were determined by atomic absorption spectrometry [21].

### 4.7. Quantitative Real-Time PCR (qRT-PCR) Analysis

RNA was extracted, and cDNA was synthesized according to the kit’s directions. The SYBR Green Master Mix was used to perform qRT-PCR on an ABI 7300 Real-Time device. Internal reference genes (*ACTIN1*) refer to this literature [31]. The expression data were calculated by 2^−ΔΔCT^. In addition, primers related to this paper are listed in Table S1, some of which refer to other publications.

### 4.8. Evaluation of Yeast Drought and Salt Tolerance

According to the characteristics of the *ZmSRG7* gene sequences and carrier pYES2 enzyme site features, yeast expression vector primers, namely, the upstream primer for 5′-TCAACCAATCTACTCGCTGCTAC-3′ (*BamH* I) and downstream primers for 5′-GAACACAAAATCAGGCGTCTTATTA-3′ (*Xba* I), were designed. A PCR was used to obtain the *ZmSRG7* sequence containing the restriction site. This was digested and purified before being ligated with pYES2 to create the recombinant vector pYES2-ZmSRG7. pYES2-ZmSRG7 and empty PY-ES2 vectors were transferred into yeast INVSC1, resulting in INVSC1 (pYES2-ZmSRG7) and INVSC1 (pYES2), with the latter serving as the control. Monoclonal yeast cells INVSC1 (pYES2) and INVSC1 (pYES2-ZmSRG7) were chosen and incubated for 12 h at 30 °C in SC-URA liquid medium containing 2% glucose. The yeasts’ body weight was collected and suspended in SC-URA liquid medium containing 2% galactose with an initial OD_600_ = 0.5. The culture was then continued at 30 °C to OD_600_ = 1.6, and the thalli were collected for stress treatment.

### 4.9. Yeast Two-Hybrid System (Y2H)

ZmSRG7’s complete open reading frame (CDS) was cloned into the c-terminus of the GAL4 DNA-binding domain in pGBKT7. Next, the CDS of interactive candidate gene *Zmdhn1* was cloned into the pGADT7 vector, and then, the recombinant plasmid ZmSRG7-BD was used as a decoy to search the STRING database (https://string-db.org/ (accessed on 1 February 2022)) for all possible interactions between the encoded proteins (Figure S2). Finally, yeast cells of AH109 were transformed with the recombinant plasmids ZmSRG7-BD and Zmdhn1-AD via the lithium acetate technique [42]. DDO culture medium SD (synthetic-defined)/-Trp/-Leu was used to test the efficacy of the transformation, while QDO medium SD/-Leu/-Trp/-His/-Ade was used to confirm the protein–protein interaction. The pGADT7-T and pGBKT7-53 constructs were used as positive controls, while the pGADT7-T and pGBKT7-lam constructs served as negative controls. Beijing Kulaibo Technology Co., Ltd.’s yeast transformation system was used as a reference throughout the yeast transformation process.

### 4.10. Statistical Analysis

All of the findings in this study were replicated three times. For statistical analysis of experimental measurement data, SPSS 24.0 software was utilized, and unidirectional ANOVA was performed to confirm the variability of results between treatments. Non-significance (ns) was set at *p* < 0.05 (**).

## Data Availability

All data generated or analyzed during this study are available within the article or upon request from the corresponding author.

## Supplementary Materials

The following supporting information can be downloaded at: https://www.mdpi.com/article/10.3390/ijms232113349/s1.
