# Supplementary material for: Overexpression of ZmSRG7 Improves Drought and Salt Tolerance in Maize (Zea mays L.)
Source: Int J Mol Sci. 2022 Nov 1;23(21):13349. doi: 10.3390/ijms232113349 (PMC9654355; doi:10.3390/ijms232113349)
Supplement: Supplementary file 1 [file ijms-23-13349-s001.zip › ijms-1941035-supplementary.pdf]

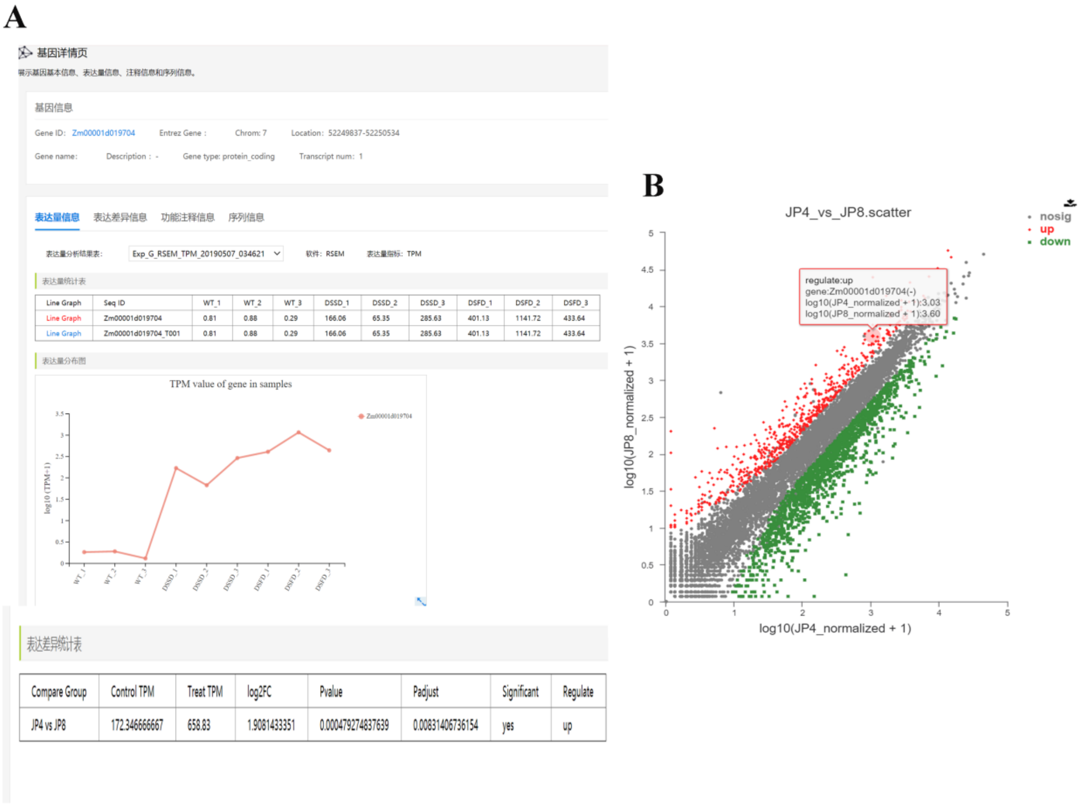

Figure S1. Transcriptome database preliminary data (NCBI: PRJNA793522). (A), Zm00001d019704 (*ZmSRG7*) gene expression distribution and statistic of difference in expression; (B), Differential scatter plot of *ZmSRG7* gene expression. The red dots represent significantly up-regulated genes, the green dots represent significantly down-regulated genes, and the gray dots represent non-significantly different genes.

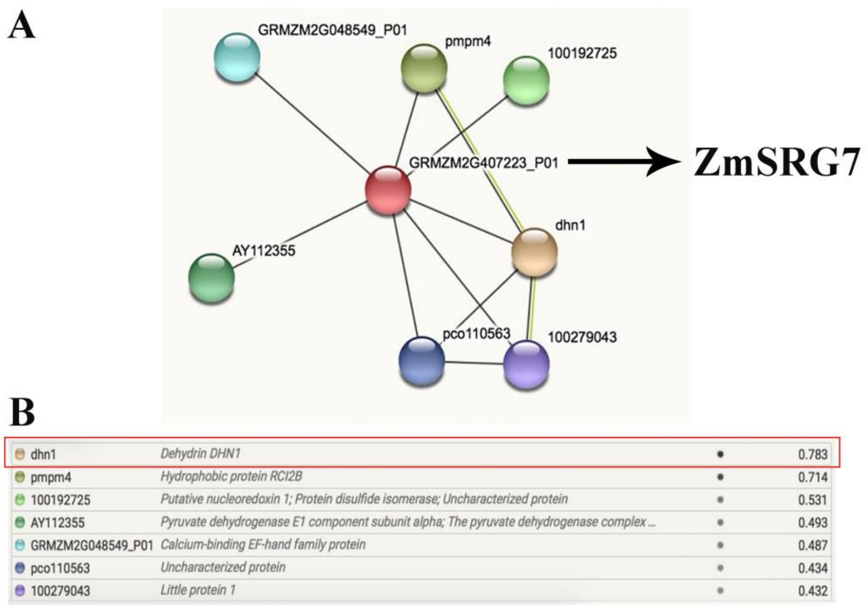

Figure S2. ***ZmSRG7* interacting proteins predicted online.** (A), ZmSRG7 protein was predicted by online software STRING (https://string-db.org/); (B), According to the findings, Zmdhn1 interaction performed the best.

Table S1. List of primers used in this study

Name	Sequence 5'-3'
ZmSRG-F	TCAACCAATCTACTCGCTGCTAC
ZmSRG-R	GAACACAAAATCAGGCGTCTTATTA
ZmActin1-F	ATGTTTCCTCCCATTGCCGAT
ZmActin1-R	CCAGTTTCGTCATACTCTCCCTTG
ZmSOS1-qPCR-F	TCATCATCCTCACAATGGCTCTAA
ZmSOS1-qPCR-R	ACCAACTTGCGTGGGACAACTTTA
ZmLTP3-qPCR-F	GGCTTTCGCTTTGAGGTTCTTC
ZmLTP3-qPCR-R	ATGCTAACACCGCACTTTCCAG
ZmSOD1-qPCR-F	TGAGGGTGTTACGGGGACTATCT
ZmSOD1-qPCR-R	GTGAAGGTGGCAGTTCCATCATCTC

ZmCAT3-qPCR-F	GCGTTGAAACCTAACCCGAAAA
ZmCAT3-qPCR-R	AAACCCTCCATGTGCCTGTAATCTT
ZmRD29B-qPCR-F	AGAGGTGGTGTAACGGGTAA
ZmRD29B-qPCR-R	GGCTCAATGGGTTTGGTG
ZmRD22-qPCR-F	GCG GGCGGGCGGCGGGCGCCT G
ZmRD22-qPCR-R	TCAGCCGCCGCGGGTCCAGACGAC G
ZmCBF4-qPCR-F	CTGG GAGGAAGAAGTTTCGTGAG
ZmCBF4-qPCR-R	CGTTATGATT CCAGCCAACTTCC
ZmABI4-qPCR-F	GACTTCGTTTCATCATGAGGTG
ZmABI4-qPCR-R	GACTTCGTTTCATCATGAGGTG
ZmCOR15-qPCR-F	CCACCGACTCCTCTCTGCTT
ZmCOR15-qPCR-R	AAGGGAGATTCCGAGATATGAAGA
ZmDREB2A-qPCR-F	GAGGACCAGAGAATAGCCGATG
ZmDREB2A-qPCR-R	GTACTCGTAACCTCAGACGCAT
ZmSnRK2.6-qPCR-F	ATGGATCGACCAGCAGTGAG
ZmSnRK2.6-qPCR-R	GCCAATATCCTTGACGAGTT
ZmNCED3-qPCR-F	CAATCATCAAACTCTCCCGCC
ZmNCED3-qPCR-R	TCTCGTGGCTGACAAGGAAAC
ZmHKT1-qPCR-F	TCGGCTCTGGACCTACTCTT
ZmHKT1-qPCR-R	ACGACGACGACTCTGCTCTA
ZmNHX1-qPCR-F	ATGTGGCGTTACGGTGCGG
ZmNHX1-qPCR-R	GGTCATTTGGTGGGCGGG
ZmSh1-qPCR-F	GGCTCATAACTACAAGGGCAC
ZmSh1-qPCR-R	TCCGAGTAGGGAGTGTCTTG
ZmSus1-qPCR-F	TGAGCTTGTCGCCGTCTTC
ZmSus1-qPCR-R	CACCATCCTTGAGCTTCTCG
